# Calcium Signaling in MR1-Dependent Antigen Presentation of Mycobacterium tuberculosis

**DOI:** 10.21203/rs.3.rs-3154465/v1

**Published:** 2023-08-30

**Authors:** Elham Karamooz, Jessie Peterson, Allison Tammen, Shogo Soma, Se-Jin Kim, David Lewinsohn

**Affiliations:** Portland VA Medical Center; Portland VA Medical Center; Oregon Health & Science University; Oregon Health & Science University; Oregon Health & Science University; Department of Pulmonary and Critical Care Medicine, Oregon Health & Science University, Portland, OR 97239, USA

## Abstract

MR1 is a ubiquitously expressed MHC-Ib molecule that presents microbial metabolites to MR1-restricted T cells, but there are differences in the antigen presentation pathway of an intracellular microbe compared to exogenous antigen. We have shown the importance of endosomal trafficking proteins in MR1-dependent presentation of *Mycobacterium tuberculosis* (Mtb). Two pore channels (TPCs) are endosomal calcium channels that regulate endosomal trafficking. Due to their location on endosomes, we hypothesized that TPCs could be required for MR1-dependent presentation of antigens derived from the intracellular microbe Mtb. We found that TPCs are critical for the presentation of Mtb by MR1; inhibition of TPCs had no effect on MR1 presentation of extracellular (exogenous) antigens, HLA-B presentation, or HLA-II presentation. Finally, we found that the calcium sensitive trafficking protein Synaptotagmin 7 was also key in the presentation of Mtb by MR1. This calcium-dependent endosomal pathway is a novel mechanism by which the immune system can sample intracellular antigens.

MR1-restricted T cells (MR1Ts) are an abundant class of CD8+ T cells with a high prevalence in the peripheral blood as well as certain organs such as the lungs [1–3]. They detect a variety of pathogens, including *Mycobacterium tuberculosis* (Mtb), and produce proinflammatory cytokines in response to these microbes [1,2]. Unlike MHC-Ia molecules, which display peptides, MR1 is an MHC-Ib molecule that displays small microbial metabolites derived from riboflavin biosynthesis and other metabolic pathways [4–6]. Our previous work established the reliance of MR1-dependent presentation of Mtb on endosomal trafficking [7]. We also described differences between MR1 presentation of Mtb, an intracellular microbe, and exogenously added antigens [8]. Critically, the endosomal trafficking proteins identified in MR1-dependent antigen presentation played no role in HLA-B antigen presentation [7,8].

The human airway epithelial cell line BEAS-2B is very effective at presenting Mtb antigens to MR1Ts despite a lower infection efficiency compared to human dendritic cells (DCs) [9]. In DCs, Mtb interference with phagolysosome maturation results in a phagosome that is positive for the early endosome marker Rab5 [10]. In contrast, in BEAS-2B, Mtb resides in a late endosomal compartment defined by Rab7 and LAMP1 [9]. When MR1 tagged with GFP is overexpressed in these cells, the MR1 vesicles also associate with Rab7 and LAMP1 [7]. Despite the commonality of endosomal markers between the MR1 and Mtb compartment in BEAS-2B, it is unclear whether MR1 is physically present in the Mtb compartment.

Calcium signaling is necessary for the proper functioning of the immune system. The intracellular calcium concentration is tightly controlled and elevation of the calcium concentration triggers cellular mechanisms such as cytokine release and proliferation [11]. Elevation of the intracellular calcium concentration can be diffuse or local. Late endosomes are rich in intracellular calcium and local calcium release regulates their trafficking [12,13]. This calcium release is governed by endosomal calcium channels, which include the mucolipins and two-pore channels (TPC) [11,13]. Inhibition of TPCs leads to impaired endosomal trafficking, which has implications for the control of intracellular infections. For example, in a mouse model of Ebola virus infection, pharmacologic inhibition of TPCs decreased infectivity and improved survival by preventing the trafficking of the virus in the infected cell [14]. Given the importance of endosomal trafficking in MR1-dependent presentation of Mtb, we hypothesized that endosomal calcium signaling would play a role in MR1-dependent presentation of Mtb.

First, we investigated whether calcium channel blockade with the L-type calcium channel blocker tetrandrine affects MR1-dependent presentation of Mtb. To measure antigen presentation, we used specific human T cell clones that produce IFN-γ in response to Mtb infected antigen presenting cells (APCs). We infected BEAS-2B with H37Rv Mtb (MOI 8). After 6 hours, cells were treated with 10uM tetrandrine or DMSO. After overnight treatment, the cells were incubated with MR1Ts or HLA-B45-restricted human T cell clones in an IFN-γ ELISpot assay. We found that tetrandrine decreased both MR1- and HLA-B45-restricted antigen presentation (Fig. 1a). The small molecule 6-FP is an MR1 antagonist derived from the photodegradation of folic acid [4]. Although unable to activate MR1Ts, 6-FP is a potent MR1 ligand that is loaded in the endoplasmic reticulum and induces MR1 translocation to the cell surface [15,16]. Using BEAS-2B stably transduced with a doxycycline inducible MR1-GFP construct (TET-MR1GFP), we found that tetrandrine decreased MR1 surface stabilization after treatment with 6-FP compared to control (Fig 1b). We also found that tetrandrine caused enlargement of MR1GFP vesicles (Fig 1c).

The effects of tetrandrine on MR1- and HLA-B45-restricted antigen presentation suggested that the spectrum of activity of tetrandrine was too broad, therefore we sought to test the role of specific endosomal calcium channels in antigen presentation. We used RT-qPCR to determine the relative amounts of TPC1 and TPC2 in BEAS-2B and primary human DCs. We found that TPCs were expressed at levels similar to or greater than MR1 in both cell types and DCs had a higher expression of MR1, TPC1 and TPC2 compared to BEAS-2B (Fig. 2a). TPCs release endosomal calcium in response to the ligand nicotinic acid adenine dinucleotide phosphate (NAADP), a process inhibited by the small molecule *trans*-Ned-19 (N19) [17,18]. To test the effect of N19 on antigen presentation, we treated BEAS-2B with 25uM N19 or DMSO 6 hours after Mtb infection and performed IFN-γ ELISpot assays. We found that N19 decreased MR1 presentation of Mtb but had no effect on HLA-B45 presentation (Fig. 2b). Next, we used exogenous antigens to test whether the effect of N19 was limited to Mtb. For MR1, we used filtered *Mycobacterium smegmatis* supernatant (Msmeg supernatant) and for the HLA-B45-restricted T cells, we used peptide CFP10_2–9_. We found that antigen presentation of exogenously added antigens was unaffected by N19 (Fig. 2c). Microscopy of BEAS-2B transduced with TET-MR1GFP showed no perturbation of the vesicles by N19 (Fig. 2d). These data indicate that TPC calcium release is important for MR1-mediated presentation of Mtb but not for HLA-B45 presentation of Mtb or any exogenously delivered antigens.

To determine if N19 impacted MR1 surface stabilization, we performed flow cytometry on TET-MR1GFP cells treated with 6-FP. We found that N19 had no effect on MR1 surface stabilization after addition of 6-FP (Fig. 3a). These data indicate that N19 does not interfere with MR1 loading in the ER or the ability of the 6-FP loaded MR1 to reach the cell surface. To determine whether N19 caused a change in MR1 transcripts in Mtb infected cells, we performed RT-qPCR on Mtb infected cells that were treated with N19 versus control (Fig. 3b). We found no difference in MR1 transcripts between the two conditions, indicating that the effect of N19 on Mtb infected cells was not due to changes in MR1 mRNA levels.

The most potent MR1 ligand is 5-OP-RU, a product of 5-A-RU and methylglyoxal [19]. Although 6-FP does not activate MR1Ts, pretreatment of APCs with 6-FP boosts T cell responses when exogenous antigens, including 5-OP-RU, are used [8]. Because 5-A-RU is unstable, a 5-A-RU prodrug was developed that requires enzymatic cleavage in acidic endosomes to form 5-A-RU [20]. To test whether N19 affected 6-FP mediated boosting of the 5-A-RU prodrug, we treated BEAS-2B with N19 or DMSO and 6-FP or 0.01M NaOH. The next day, the cells were used in an IFN-γ ELISpot assay and additional N19 was added into the ELISpot wells so that N19 would be present with the 5-A-RU prodrug and the MR1Ts. We found that N19 had no effect on 5-A-RU prodrug presentation or the boosting effect from 6-FP pretreatment (Fig. 3c).

The exclusive effect of N19 on MR1 presentation of Mtb raised the possibility that N19 could affect Mtb uptake or Mtb viability. Either mechanism would lead to a decrease in the quantity of Mtb antigens available to MR1, and it was shown that N19 can affect small and large particle uptake in short assays of up to 90 minutes [21]. While N19 did not affect the processing or presentation of Mtb derived CFP10 (Fig 2c, left), we sought to directly test whether N19 affected Mtb uptake or Mtb viability. We performed growth assays on Mtb infected BEAS-2B treated with N19 and found a mean difference of 8.23 Mtb colony forming units (CFU) per 100 cells, which was not statistically significant. (Fig. 3d). These data are consistent with the Mtb/HLA-B45 assays, which showed no effect from N19.

Although BEAS-2B present Mtb to MR1Ts, they are not professional APCs and the mechanisms underlying myeloid presentation of Mtb likely differ from those of airway epithelial cells. To determine whether the effect of N19 was generalizable to professional APCs, we treated primary human DCs with 100uM N19 or DMSO 6 hours after Mtb infection and performed IFN-γ ELISpot assays. We found that N19 affected MR1-dependent presentation of Mtb in DCs (Fig. 4a). There was no effect on MR1-dependent presentation of exogenous antigens (Fig. 4a). Since DCs have HLA-II, which has a well-established endosomal trafficking component, we characterized the effect of N19 on HLA-II presentation of Mtb using an HLA-II-restricted T cell clone that detects CFP10 [10]. An HLA-B8-restricted T cell clone was used as a control. We found that N19 had no effect on HLA-II or HLA-B8 presentation in DCs (Fig. 4b,c). These data show that in DCs, N19 specifically affects MR1 presentation of Mtb without impacting HLA-II, HLA-B8 or exogenous antigen presentation.

In humans, there are two TPC proteins, TPC1 and TPC2 [12,13]. To confirm that the effect of N19 was due to inhibition of TPCs, we first used the TPC2 inhibitor YM201636 [22]. Inhibition of TPC2 in BEAS-2B had no effect on MR1 or HLA-B45 antigen presentation of Mtb or exogenous antigens (Fig 5a,b). Since a TPC1 inhibitor is not available, we performed siRNA knockdown of TPC1 in BEAS-2B. Knockdown of TPC1 reduced TPC1 transcripts by 76% in 48hrs (Fig 5c). Functionally, TPC1 knockdown resulted in a significant reduction in MR1-mediated presentation of Mtb and a small effect on MR1 presentation of exogenous antigens (Fig. 5d,e). However, there was no significant effect on HLA-B45 presentation of Mtb or exogenous antigen (Fig. 5d,e).

Since TPC-mediated calcium release facilitates endosomal trafficking, and TPC1 is associated with recycling and late endosomes [23.24], we hypothesized that calcium-sensitive endosomal trafficking proteins play a role in MR1 presentation of Mtb. Synaptotagmin 7 (Syt7) is a calcium sensitive endosomal trafficking protein implicated in LAMP1 delivery to phagosomes and translocation of MHC-II to the plasma membrane [25,26]. To test the role of Syt7 in MR1-dependent antigen presentation, we knocked down Syt7 using siRNA, resulting in an 86% reduction in Syt7 transcripts (Fig 5f). Functionally, Syt7 knockdown affected MR1-dependent presentation of Mtb without a significant effect on MR1 presentation of exogenous antigens (Fig. 5g). Taken with the TPC1 knockdown experiments, these data show that TPC1 and Syt7 knockdown have MR1-Mtb specific effects consistent with N19 treatment.

McWilliam and colleagues established the importance of the ER MR1 antigen presentation [16]. In this model, a preformed pool of MR1 is retained in the ER. After acquiring a ligand that forms a Schiff base with lysine 43 of MR1, MR1 associates with β_2_M and translocates to the cell surface [16]. This has been demonstrated with exogenously added ligands such as Acetyl-6-FP and 5-OP-RU. In the setting of *Salmonella enterica* serovar Typhimurium infection, MR1-dependent antigen presentation was substantially reduced by Brefeldin A, which prevents protein egress from the ER [16]. Follow-up studies using a fluorescent MR1 ligand confirmed that the loading occurred in the ER [27]. In contrast, our studies focus on the mechanism of MR1 presentation of antigens derived from Mtb. We previously found that certain endosomal trafficking proteins played a role in MR1 antigen presentation and elucidated differences in antigen presentation pathways between intracellular Mtb infection and exogenously added mycobacterial antigens [7,8]. Specifically, we showed that pretreatment with the inhibitory ligand 6-FP boosted presentation of exogenously added antigens but had no such effect with Mtb infection [8]. Furthermore, we found that Syntaxin 4 knockdown inhibited exogenous antigen presentation by MR1 but had no effect on Mtb presentation [8]. These results implied that the MR1 antigen presentation pathway differed for Mtb compared to exogenous antigens.

In this study, we expand on our earlier work and definitively show that the mechanism of MR1 presentation of intracellular Mtb infection differs from exogenous antigen presentation in human APCs. First, we show that treatment with the L-type calcium channel blocker tetrandrine decreased both MR1- and HLA-B45-dependent presentation of Mtb. Next, we investigated endosomal calcium channels given our prior work on the importance of endosomal trafficking in MR1. Since late endosomes are rich in calcium and express TPCs [12], we targeted TPCs using N19, which inhibits NAADP-mediated calcium release from TPCs [18]. We found a significant and specific decrease in Mtb antigen presentation to MR1Ts in both human airway epithelial cells and primary human DCs. There was no effect on exogenous antigen presentation, further supporting that the mechanisms of MR1 presentation of Mtb differs from that of exogenously added antigens. It is unlikely that the mechanism of N19 inhibition of MR1-Mtb presentation stems from blocking of acidification of the endosome because N19 had no effect on the 5-A-RU prodrug, which requires an acidic compartment for cleavage into 5-A-RU [20].

Previous work indicated that TPCs are important for the phagocytic function of murine bone marrow-derived macrophages [21]. In that work, uptake of silica beads, *Mycobacterium smegmatis* and an attenuated form of *Mycobacterium bovis* (BCG) were used to measure phagocytic function. However, uptake was measured at a maximum of 90 minutes. Given those results, we performed orthogonal assays to determine whether our findings were due to impaired Mtb uptake or unexpected toxicity from N19. First, we used an HLA-B45 restricted T cell clone that detects CFP10_2–9_ from Mtb. Results showed no significant effect of N19 on HLA-B45 antigen presentation, indicating a similar amount of internalized Mtb antigen between the groups. Additionally, we performed growth assays between the treatment and control groups and found no significant difference in the number of viable Mtb between conditions. These data argue that the results with N19 did not reflect impaired uptake or mycobacterial viability.

The results obtained with N19 were confirmed with TPC1 knockdown. We then evaluated the role of the calcium sensitive endosomal trafficking protein Syt7 and found that it is also key to MR1-dependent presentation of Mtb. Mechanistically, there are different plausible roles for TPC1 and Syt7 in MR1-dependent antigen presentation. TPC1 is expressed in different organs, including in the lungs, and colocalizes with recycling endosomes, endolysosomes and late endosomes [23,24]. Therefore, TPC1 could be responsible for local endosomal calcium release that activates Syt7. However, in HeLa cells and primary human fibroblasts, TPC1 is required for the formation of late endosome-ER contact sites [28]; both N19 and TPC1 knockdown reduced the number of contacts between late endosomes and the ER [28]. Therefore, it is possible that TPC1 mediated endosome-ER contact sites could deliver MR1 antigens to the ER. Regarding the mechanism of Syt7 in MR1-dependent antigen presentation, at least two possibilities exist. First, Syt7 functions in the early recruitment of LAMP1 to the phagosome in murine bone marrow derived macrophages [25]. In human DCs and BEAS-2B, the percentage of Mtb positive for LAMP1 was 90% and 58%–80% (depending on the time point), respectively [9,29]. Thus, it is possible that Syt7 knockdown interferes with LAMP1 delivery to the Mtb compartment and that this is necessary for MR1 presentation of Mtb. A second possibility is that Syt7 shuttles loaded MR1 to the cell surface. This is mechanism is based on Syt7 knockout mice, where DCs had decreased in MHC-II surface expression [26].

In conclusion, we have identified a novel mechanism for antigen presentation of Mtb that utilizes endosomal calcium signaling. This calcium-sensitive pathway is specific to MR1 and had no role in HLA-B or HLA-II antigen presentation. At present, specific Mtb antigens for MR1 are not known, making it difficult to track the acquisition and delivery of antigens intracellularly. Despite these limitations, our data show a pathway, independent of exogenous antigen delivery, in which endosomal calcium signaling and calcium sensitive trafficking proteins are essential for MR1-dependent presentation of Mtb.

## Methods

### Bacteria and cells

*Mycobacterium tuberculosis* (Mtb) H37Rv (ATCC) was grown in Middlebrook 7H9 broth supplemented with Middlebrook ADC, 0.05% Tween-80, and 0.5% glycerol. The bacteria were passaged 10–20 times through a tuberculin syringe before infection. Multiplicity of infection of 8 was used for all Mtb experiments. Mtb growth assays were performed by lysing 200,000 infected BEAS-2B in ultrapure water. Serial dilutions were performed with PBS+0.05% Tween 80 and lysates were plated on 7H10 plates supplemented with glycerol and Middlebrook ADC. Plates were incubated at 37 C and 5% CO_2_.

*Mycobacterium smegmatis* (Msmeg) supernatant was passed through a 0.22 μm filter and concentrated across a 10kDa Amicon filter (Millipore Sigma). The supernatant was aliquoted and stored at −80C. All experiments with Mtb were done in a Biosafety Level 3 laboratory. Waste was decontaminated in 3% Wescodyne and autoclaved. All other experiments were performed in a Biosafety Level 2 laboratory.

BEAS-2B (ATCC) and BEAS-2B transduced with TET-MR1GFP [30] were cultured in DMEM (Gibco) supplemented with L-glutamine and 10% heat inactivated fetal bovine serum (FBS). Human dendritic cells (DCs) were prepared from peripheral blood mononuclear cells [31]. Briefly, PBMC were resuspended in 10% heat inactivated human serum in RPMI (Gibco) supplemented with L-glutamine (Gibco), gentamicin (Gibco) and DNAse (Roche), and placed in a T-75 flask and allowed to adhere at 37C with 5% CO2. After 1 hour, the flask was gently rocked and nonadherent cells removed. The cells were treated with 300ng GM-CSF (Sanofi) and 300ng IL-4 (R&D Systems). The flask was irradiated with 3000 cgray and DCs were used on day 5. The following human T cell clones were used: TRAV1–2+ MR1-restricted (D426-G11) [5], HLA-B45-restricted (D466-A10, minimal epitope CFP10_2–9_) [32], HLA-B8-restricted (D480-F6, minimal epitope CFP10_3–11_) [10], and HLA-II restricted (D454-E12, detects CFP10_25–39_) [10].

### Reagents and antibodies

Tetrandrine (Selleck Chemicals) was obtained as a 10mM stock in DMSO and used at 10μM. *Trans*-Ned-19 (N19, Cayman Chemical) was resuspended to 2.5mg/mL in DMSO and used at 25μM for BEAS-2B and 100μM in DCs. YM-201636 (MedChemExpress) was obtained as a 10mM stock in DMSO and used at 5μM. 6-formylpterin (Schirck’s Laboratories) was resuspended at 1mg/mL in 0.01M NaOH and used at 100μM. Doxycycline (Sigma-Aldrich) was suspended at 2 mg/mL in sterile water and used at 2 μg/mL. 5-A-RU prodrug (**5-A-RU-PABC-Val-Cit-Fmoc,** MedChemExpress) was resuspended in DMSO at 10mM. Phytohemagglutinin (PHA, Sigma-Aldrich) was resuspended to 10mg/mL in 10% human serum in RPMI (Gibco) supplemented with L-glutamine and gentamicin. Paraformaldehyde (Electron Microscopy Sciences) was obtained as a 16% stock and diluted to a 4% working stock using phosphate buffered saline (Corning). Anti-MR1 mouse monoclonal antibody APC (Clone 26.5; BioLegend) and isotype control antibody mouse IgG2a (BioLegend) were used at 1:166. FBS (GeminiBio) was heat inactivated at 56C for 45 minutes. Human serum was obtained from donors and heat inactivated.

### ELISpot assays

ELISpot assays were done using 96 well MSHA plates (Merck Millipore) and coated with an antibody against IFN-γ (Mabtech). Antigen presenting cells were harvested using 10% human serum in RPMI (Gibco) supplemented with L-glutamine and gentamicin. The cells were resuspended to 2e5 for Mtb infected BEAS-2B and 1e5 for DCs and uninfected BEAS-2B. The cells were plated at 100μL/well in the MSHA plates in duplicate. For Mtb experiments with tetrandrine, N19, or YM-201636, cells were treated with drug 6 hours after Mtb infection. For all Mtb infected APCs, serial dilutions were performed. For uninfected APCs, the APCs were kept constant and Msmeg supernatant or peptide was added as a serial dilution with the final volumes kept constant. PHA was used as a positive control for all ELISpot assays (1μg/well). After 1 hour, T cells were added at 1e4 cells/well. After overnight incubation at 37C and 5% CO_2_, the plate was developed with ALP antibody (Mabtech). IFN-γ spot forming units (SFU) or IFN-γ cytokine activity were measured on an AID ELISpot reader. The mean of technical replicates was used to pool data from different experiments.

ELISpot assays using the 5-A-RU prodrug were done with BEAS-2B stably transduced with TET-MR1GFP. The cells were cultured in a 6 well plate and MR1GFP was induced with 2μg/mL doxycycline. The next day, the cells were treated with 25uM N19 versus DMSO and 100uM 6-FP versus 0.01M NaOH. The next day, the cells were harvested and resuspended to 1e5 cells/mL and re-treated with N19 versus DMSO, then plated at 100μL/well (1e4 cells) in duplicate (final concentration of N19 was 25μM in the ELISpot well). 5-A-RU prodrug was diluted to 1uM in 10% human serum and RPMI supplemented with L-glutamine and gentamicin. 5-A-RU prodrug was added at 50μL/well and serial dilutions performed (starting concentration in the wells was 0.25uM. After 1 hour, T cells were added at 1e4 cells/well (50μL/well) resulting in a final volume of 200μL/well.

### Flow cytometry

BEAS-2B transduced with TET-MR1GFP were induced with doxycycline in a 6 well plate (Corning). The next day, the cells were treated with drug versus DMSO as well as 100uM 6-FP versus 0.01M NaOH. The next day, the cells were stained for surface MR1 versus isotype control in 2% human serum, 2% goat serum, and 0.5% FBS on ice. After 40 minutes, the cells were washed, fixed in 1% PFA and analyzed with a BD LSR II flow cytometer. All analyses were performed using FlowJo software (BD).

### Microscopy

BEAS-2B transduced with TET-MR1GFP were plated into a 4-well 1.5 mm glass bottom chamber slides (Nunc) and incubated at 37C and 5% CO2. After 24 hrs of doxycycline treatment, the cells were treated with test drug (tetrandrine or N19) versus DMSO control. The next day, the cells were imaged live on a DeltaVision Wide field Deconvolution microscope with a 60x objective (NA 1.42) and a Nikon Coolsnap ES2 HQ. Each image was acquired as Z-stacks in a 1024×1024 format. Images were processed on Imaris (Bitplane).

### siRNA knockdown

BEAS-2B plated in 6-well tissue culture plates (Corning) at 70% confluency were transfected with 50nM Silencer Select siRNA (Missense Negative Control #1, TPCN1 s28727 or SYT7 s17292) using HiPerFect (Qiagen). Knockdown was done for 48 hours. For functional assays, the knockdown cells were infected with Mtb or left uninfected for exogenous antigens, and used the following day in an IFN-γ ELISpot assay.

### RNA isolation, cDNA synthesis and RT-qPCR analysis

Total RNA was isolated using RNeasy Mini Kit (Qiagen). cDNA was synthesized using a High Capacity cDNA Reverse Transcription Kit (ThermoFisher Scientific). RT-qPCR was performed using TaqMan Universal PCR Master Mix (ThermoFisher Scientific) on a Step One Plus Real-Time PCR System (Applied Biosystems). FAM-MGB TaqMan Gene Expression Assays for all targets were obtained from ThermoFisher Scientific. Reactions were run in triplicate and data normalized to GAPDH. Expression levels were determined using the 2^−ΔΔCT^ method.

### Ethics statement

This study was conducted according to the principles expressed in the Declaration of Helsinki. Study participants, protocols and consent forms were approved by Oregon Health & Science University Institutional Review Board (IRB00000186). Written and informed consent was obtained from all donors.

### Data analysis

Data were analyzed with GraphPad Prism 9.5. All ELISpot assays were plated with technical replicates. Mean data from technical replicates were pooled from 3 independent experiments. Nonlinear regression was done using agonist vs. response (3 parameters) and a bottom constraint set to 0. For flow cytometry, data were pooled from 3 independent experiments. For all *t* tests, a paired, two-tailed analysis was performed.

## Figures and Tables

**Figure 1:**
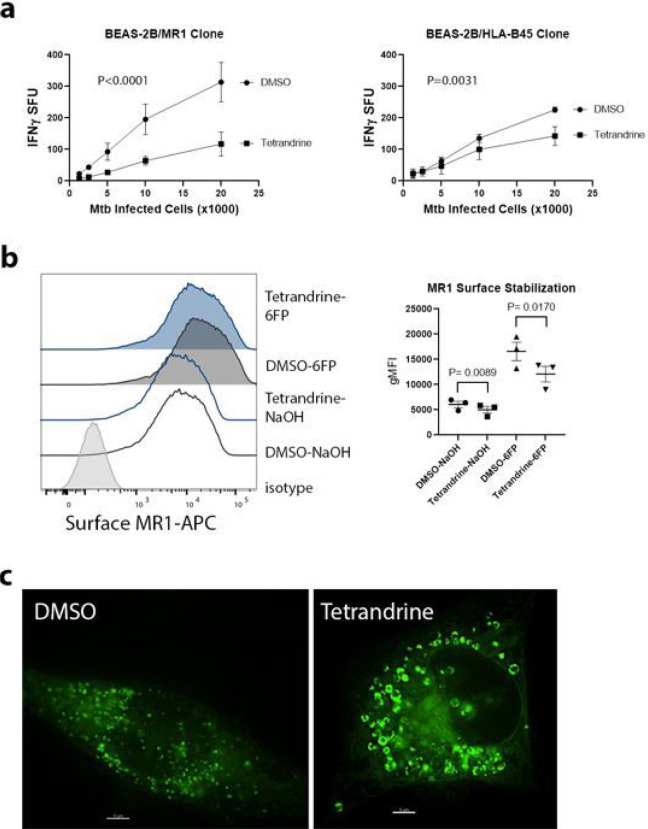
Tetrandrine decreases Mtb antigen presentation. a) IFN-γ ELISpot assays in BEAS-2B infected with Mtb and treated with 10μM tetrandrine or DMSO. Cells were incubated with MR1Ts (left) or HLA-B45-restricted (right) T cell clones. Mean values from technical replicates were pooled from 3 independent experiments (mean and SEM graphed) and nonlinear regression analysis performed. b) Effect of tetrandrine on MR1 surface stabilization in BEAS-2B transduced with BEAS-2B expressing doxycycline inducible MR1-GFP (TET-MR1GFP) treated with 100μM 6-FP or 0.01M NaOH. Representative histogram (left) and gMFI from 3 independent experiments (right). P values from paired, two-tailed *t* tests. c) Effect of tetrandrine on MR1-GFP vesicles in BEAS-2B transduced with TET-MR1GFP. Representative images from 3 independent experiments. Scale bars represent 5μm.

**Figure 2:**
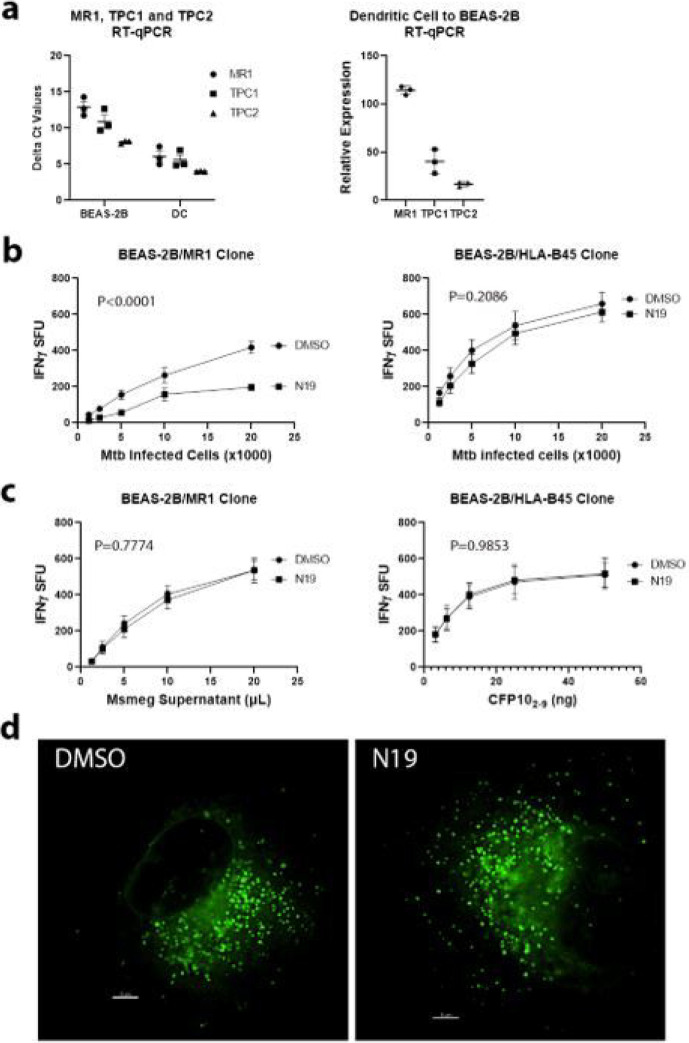
TPC blockade specifically impairs MR1 presentation of Mtb. a) Comparison of delta Ct values from RT-qPCR of MR1, TPC1, and TPC2 in BEAS-2B and DCs. Data are compared to GAPDH (left). Lower delta Ct values indicate higher expression. Relative expression of MR1, TPC1, and TPC2 in DCs compared to BEAS-2B using RT-qPCR (right). b) IFN-γ ELISpot assays of effect of N19 on MR1 and HLA-B45 antigen presentation of Mtb. c) IFN-γ ELISpot assays of effect of N19 on MR1 and HLA-B45 presentation of exogenous antigens. b-c) Mean values from technical replicates were pooled from 3 independent experiments (mean and SEM graphed) and nonlinear regression analysis performed. d) Comparison of MR1-GFP vesicles in BEAS-2B expressing TET-MR1GFP treated with N19 or DMSO. Representative images from 3 independent experiments. Scale bars represent 5μm.

**Figure 3:**
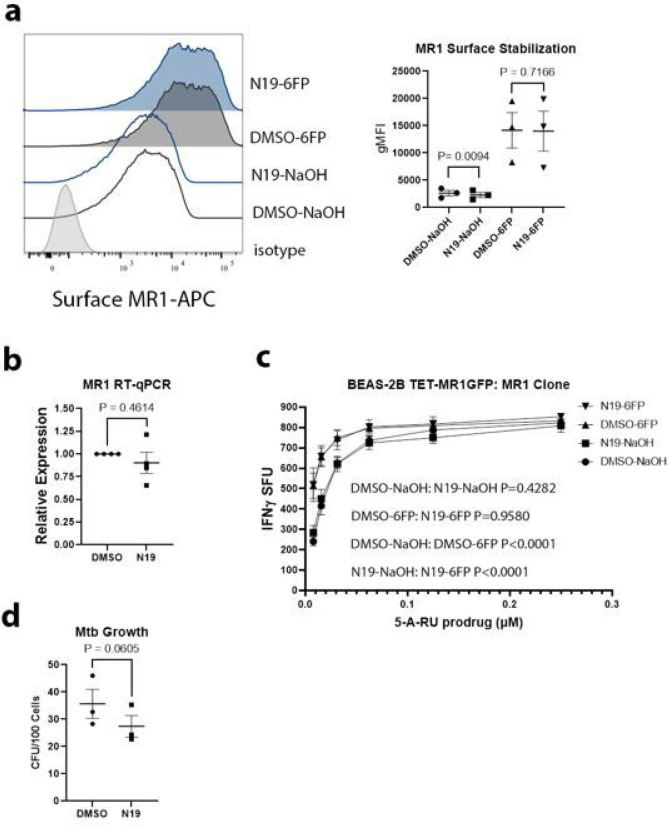
TPC blockade does not affect MR1 surface stabilization or Mtb uptake. a) effect of N19 on MR1 surface stabilization in BEAS-2B expressing TET-MR1GFP treated with 100μM 6-FP versus 0.01M NaOH. Representative histogram (left) and gMFI from 3 independent experiments (right). P values from paired, two-tailed *t* tests. b) RT-qPCR of MR1 transcripts in BEAS-2B infected with Mtb and treated with N19 versus DMSO. Data from 4 independent experiments plotted with mean and SEM. P value from paired, two-tailed *t* test. c) effect of N19 on presentation of 5-A-RU prodrug and 6-FP boosting in an IFN-γ ELISpot assay. Mean values from technical replicates were pooled from 3 independent experiments (mean and SEM graphed) and nonlinear regression analysis performed. d) colony forming units from BEAS-2B infected with MTb and treated with N19 versus DMSO. Mean from triplicates plotted from 3 independent experiments with SEM. P value from paired, two-tailed *t* test.

**Figure 4:**
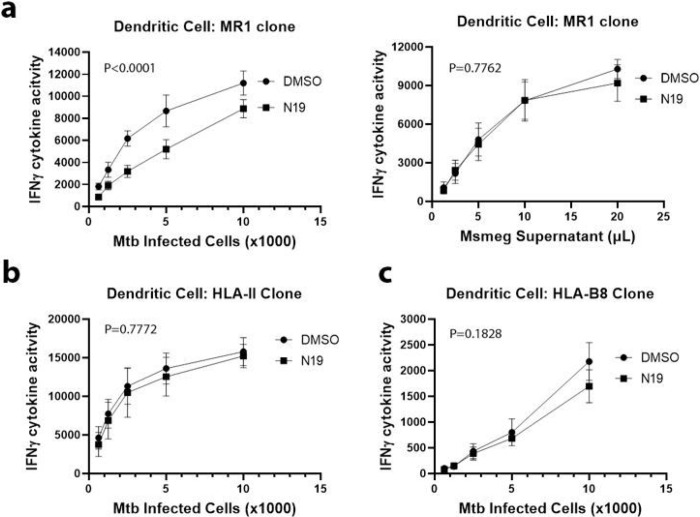
TPC blockade impairs MR1 presentation of Mtb in DCs. a) IFN-γ ELISpot assays of DCs treated with 100μM N19 and incubated with MR1Ts. Antigen presentation of Mtb (left) versus Msmeg supernatant (right). b) IFN-γ ELISpot assays with HLA-II T cell clone and Mtb infected DCs treated with 100μM N19. c) IFN-γ ELISpot assays with HLA-B8 T cell clone and Mtb infected DCs treated with 100μM N19. a-c) Mean values from technical replicates were pooled from 3 independent experiments (mean and SEM graphed) and nonlinear regression analysis performed.

**Figure 5:**
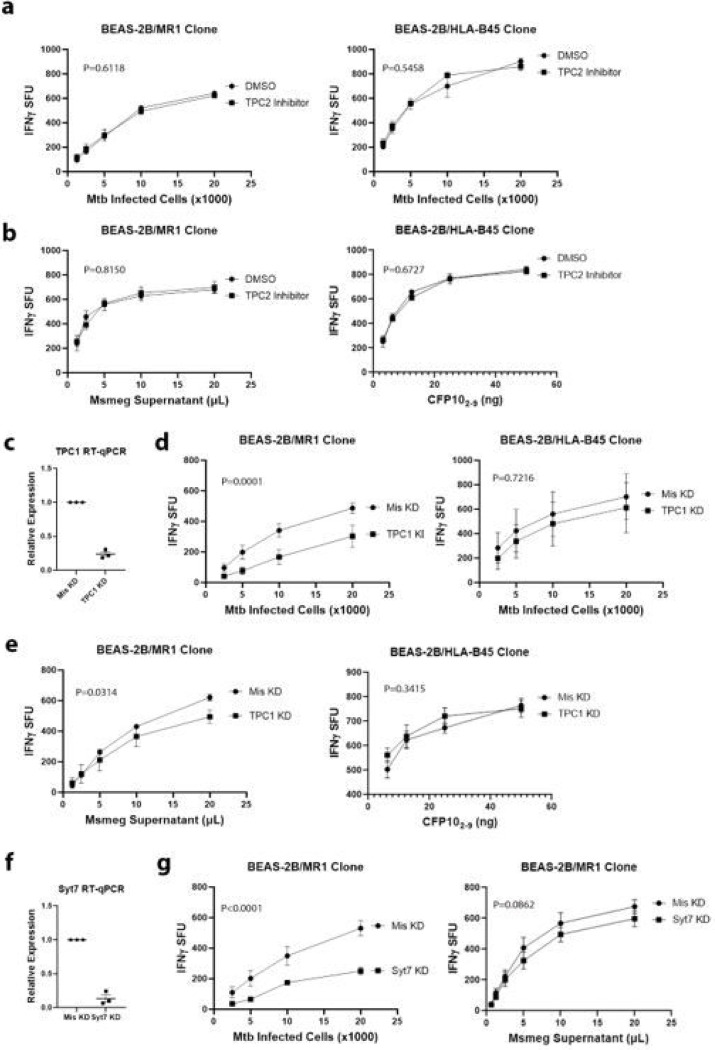
Knockdown of TPC1 and Synaptotagmin 7 specifically impair MR1 presentation of Mtb. a) IFN-γ ELISpot assays measuring effect of 5μM YM201636 on MR1 and HLA-B45 presentation of Mtb. b) IFN-γ ELISpot assays of BEAS-2B presentation of exogenous antigen by MR1 and HLA-B45 after treatment with 5μM YM201636. a-b) Mean values from technical replicates were pooled from 3 independent experiments (mean and SEM graphed) and nonlinear regression analysis performed. c) RT-qPCR of TPC1 siRNA knockdown from 3 independent experiments. Mean and SEM graphed d) IFN-γ ELISpot assays of Mtb infected TPC1 knockdown BEAS-2B using MR1- (left) and HLA-B45-restricted T cells (right). e) IFN-γ ELISpot assays of MR1 and HLA-B45 presentation of exogenous antigen by BEAS-2B after TPC1 knockdown. d-e) Mean values from technical replicates were pooled from 3 independent experiments (mean and SEM graphed) and nonlinear regression analysis performed. f) RT-qPCR of Syt7 siRNA knockdown from 3 independent experiments. Mean and SEM graphed. g) IFN-γ ELISpot assays of Synaptotagmin 7 knockdown BEAS-2B with Mtb infection (left) versus Msmeg supernatant (right). Mean values from technical replicates were pooled from 3 independent experiments (mean and SEM graphed) and nonlinear regression analysis performed.

## Data Availability

The authors declare that the data supporting the findings of this study are available within the article.
